# TNF Stimulates Nuclear Export and Secretion of IL-15 by Acting on CRM1 and ARF6

**DOI:** 10.1371/journal.pone.0069356

**Published:** 2013-08-07

**Authors:** Suidong Ouyang, Hung Hsuchou, Abba J. Kastin, Weihong Pan

**Affiliations:** Pennington Biomedical Research Center, Baton Rouge, Louisana, United States of America; University of Toronto, Canada

## Abstract

Interleukin (IL)-15 is a ubiquitously expressed cytokine that in the basal state is mainly localized intracellularly, including the nucleus. Unexpectedly, tumor necrosis factor-α (TNF) time-dependently induced nuclear export of IL-15Rα and IL15. This process was inhibited by leptomycine B (LMB), a specific inhibitor of nuclear export receptor chromosomal region maintenance 1 (CRM1). In the presence of TNF, LMB co-treatment led to accumulation of both IL-15Rα and IL-15 in the nucleus of HeLa cells, suggesting that CRM1 facilitates nuclear export and that TNF enhances CRM1 activity. Once in the cytoplasm, IL-15 showed partial co-localization with late endosomes but very little with other organelles tested 4 h after TNF treatment. IL-15Rα showed co-localization with both early and late endosomes, and to a lesser extent with endoplasmic reticulum and Golgi. This indicates different kinetics and possibly different trafficking routes of IL-15 from its specific receptor. The TNF-induced secretion of IL-15 was attenuated by pretreatment of cells by brefeldin A that inhibits ER-to-Golgi transport, or by use of domain negative ADP-ribosylation factor 6 (ARF6) that interferes with exocytotic sorting. We conclude that TNF abolishes nuclear localization of IL-15 and IL-15Rα by acting on CRM1, and it facilitates exocytosis of IL-15 with the involvement of ARF6.

## Introduction

IL-15, a 14 kD cytokine, plays an essential role in inflammatory and immune reactions. The effects of IL-15 are exerted by binding to its specific receptor IL-15Rα, which recruits the co-receptors IL-2Rβ and IL-2Rγ to initiate diverse cellular signaling events [1], [2]. IL-15Rα often exists in the same cells that produce IL-15, leading to the question whether IL-15Rα can serve as a chaperon protein for its own ligand [3].

The synthesis and secretion of IL-15 are controlled both at transcriptional and post-transcriptional levels [4]. It has a complex intracellular trafficking system that is driven by several signal peptides and alternative splicing of the coding regions for mature IL-15 [5]. Unlike most other cytokines, IL-15 exists in both secretory and intracellular forms [6]. Under basal conditions, blood concentrations of IL-15 are low and the secretory form is sparse [7]. Proinflammatory stimuli, such has lipopolysaccharide and tumor necrosis factor α (TNF), increase the production of IL-15 and its receptor [8], [9].The complex intracellular trafficking patterns might serve to curtail the potency of IL-15 as an inflammatory cytokine, or enable an efficient recruitment of intracellular pools by rapid trafficking upon stimulation.

IL-15 can localize to the nucleus; however, it does not appear to have the capability to enter the nucleus by itself [10]. Pre-pro-IL-15 contains no nuclear translocation sequence, so that a chaperon protein is needed. IL-15Rα appears to be the most suitable chaperon, as it binds to IL-15 with high affinity, and the ligand binding Sushi domain also has a nuclear localization signal [11]. The heavy O-glycosylation of IL-15Rα suggests that it “passes through” the Golgi before entering the nucleus, where it can “pick up” IL-15. The findings suggest that IL-15Rα might be a major regulator of IL-15 distribution in different intracellular organelles.

Though IL-15Rα possesses a classical nuclear localization signal, it is found both in the nucleus and cytoplasm [12]. The mechanism regulating the subcellular distribution of IL-15Rα, however, is not yet clear. Here, we hypothesize that activities of chromosome region maintenance (CRM1)/exportin 1 may be responsible for the subcellular localization of IL-15Rα, based on protein sequence analyses. CRM1 is a nuclear export receptor [13], [14]. To participate in protein nuclear export, CRM1 interacts with Ras-like nuclear G-protein GTPase, and this complex binds to the nuclear pore to translocate proteins that contain a nuclear export signal (NES) [15]. IL-15Rα contains a NES sequence which might enable its interaction with CRM1, Leptomycin B (LMB), an antifungal fatty acid that inhibits NES-dependent nuclear export by specific binding to the CRM1 [16], [17], was used to test whether its inhibition of CRM1 reduces the nuclear export of IL15-Rα. Once IL-15Rα is exported from the nucleus, other sorting signals are required to direct it to its next destination. We focused on ADP-ribosylation factor 6 (ARF6), a small GTPase known to regulate endosomal trafficking and actin dynamics [18]–[20]. Overall, identification of new interacting partners in the nuclear export of the IL-15/IL-15Rα complex and the exocytotic regulation of IL-15 not only provide mechanistic insight into the complex IL-15 system, but also illustrate the dynamic regulation of cellular inflammatory conditions such as that induced by TNF.

## Materials and Methods

### Plasmids and antibodies

ARF6-wildtype (WT) and ARF6-T27N dominant negative (DN) plasmids with human influenza hemagglutinin (HA) tag were obtained from Addgene (Cambridge, MA). To generate the IL-15Rα-GFP fusion protein plasmid, the full length IL-15Rα in pcDNA3.1(-) that was generated in our laboratory [21] was excised (*Nhe*I and *BamH*I) and subcloned into a pEGFP-N3 vector. Goat anti IL-15 antibody (Ab) (SC-1296 ), goat anti-IL-15Rα Ab (SC-1524), rabbit anti-IL-15 Ab (SC-788900), rabbit anti-IL-15Rα (SC-9172) Ab, rabbit anti-calnexin Ab (SC-6465) and goat anti Rab7 Ab (SC-6563), were purchased from Santa Cruz Biotechnology (Santa Cruz, CA); Rabbit-β-COP Ab and rabbit-EEA Ab were obtained from ABR (Golden, CO); Rabbit anti-Arf6 Ab was purchased from Proteintech Group (Chicago, IL). Mouse anti-histone Ab (MAB3422) which recognizes five histones (H1, H2A, H2B, H3 and H4) was purchased from EMD Millipore (Billerica, MA). Mouse anti-β-actin Ab and rabbit anti-HA Ab were obtained from Sigma (St. Louis, MO). All fluorescent secondary antibodies were purchased from Invitrogen Co (Grand Island, NY). Goat anti-rabbit horseradish peroxidase (HRP) Ab and goat anti-mouse HRP Ab were obtained from Jackson ImmunoResearch Laboratory (West Grove, PA).

### Cell culture, transfection and drug treatment

HeLa cells were grown in Dulbecco's Modified Eagle Medium (DMEM, Gibco, Grand Island, NY) supplemented with 10% fetal bovine serum (FBS, Gibco) and 1% penicillin-streptomycin (Gibco) at 37°C in a 5% CO_2_ incubator. HeLa cells were transfected with lipofectamine 2000 reagent (Invitrogen), following the manufacturer's protocol, and processed for immunocytochemistry (ICC) or immunoprecipitation 48 h later. In experiments to test the effects of TNF on the trafficking patterns of IL-15 or IL-15Rα, HeLa cells were treated with TNF (5 ng/mL, R&D Systems, Minneapolis, MN)) from 15 min to 4 h. In experiments to determine the involvement of cellular organelles in the trafficking process, the cells were incubated with LMB (20 ng/mL, Sigma) or brefeldin A (BFA, 2 μM, Sigma) for 2 h. LMB is a specific inhibitor of the nuclear export receptor CRM1. BFA inhibits transport of proteins from ER to Golgi and induces retrograde protein transport from the Golgi apparatus to the ER. Cell viability was determined by use of the ViaLight ^TM^ plus kit (Lonza, Rockland, ME) that monitors cellular ATP production.

### Immunocytochemistry and fluorescence microscopy

Cells were cultured on coverslips in 24-well plates. After TNF (5 ng/mL) treatment, cells were fixed with 2% paraformaldehyde (PFA) in phosphate-buffered saline (PBS) for 30 min, permeabilized with 0.1% Triton X-100 for 5 min, blocked with 10% normal donkey serum in PBS for 30 min, and then incubated with primary antibody overnight. Primary antibodies were all diluted (1∶200) in PBS containing 1% bovine serum albumin (BSA). The cells were then washed three times with PBS and incubated with appropriate secondary antibodies, including Alexa 488-conjugated donkey anti-goat Ab, Alexa 594-conjugated donkey anti-goat Ab, Alexa 488-conjugated donkey anti-rabbit Ab, or Alexa 594-conjugated donkey anti-rabbit Ab (all 1∶250) for 1 h at room temperature. After through washes, the coverslips were mounted with Prolong® Gold antifade reagent with DAPI (Invitrogen, Carlsbad, CA). Immunofluorescence was captured on an Olympus FV1000 inverted laser scanning microscope in the laboratory. The co-localization of the two channels was quantified and presented as Pearson's Coefficient and Manders' Coefficients by analysis of the confocal images by use of NIH Image J with JACoP plug-in [22] followed by manual thresholding.

### Subcellular fractionation

To isolate subcellular fractions, we used NE-PER nuclear and cytoplasmic extraction reagents (Pierce, Rockford, IL). HeLa cells (2×10^5^ per well) were grown overnight in 24-well plates to full confluency. The cells were then treated with TNF, LMB, or vehicle according to the group design detailed in the Results section and harvested with trypsin-EDTA (Invitrogen, Grand Island, NY). The cells were suspended in ice-cold 200 μL CERI solution with protease inhibitor cocktail (Sigma), and incubated on ice for 10 min. After addition of 11 μL of CERII solution and vigorous mixing by vortex for 5 sec, the cells were incubated on ice for 1 min and centrifuged at 16,000 g at 4°C for 10 min. The supernatant was kept as “cytoplasm”. The pellet was suspended with 100 µL NER solution in the presence of protease inhibitors and vortexed at the highest setting for 15 sec. After 40 min incubation on ice, the pellet was centrifuged at 16000 g for 10 min at 4°C. The supernatant was collected as “nuclei”.

### Immunoprecipitation

HeLa cells (0.5×10^6^/mL) were seeded in a 6- well plate (2 mL/well) and transfected with HA-ARF6 plasmid in pcDNA3.1. At 48 h, cells were washed with PBS and fixed with 1% PFA for 30 min at room temperature. The cross-linking reaction was stopped by incubation with 125 mM final concentration of glycine for 10 min. Cells were then harvested and lysed with 1mL of lysis buffer, centrifuged at 16000 g for 15 min. The supernatant was collected and protein concentrations were quantified with a bicinchoninic acid (BCA) protein assay kit (Pierce, Rockford, IL). Cell lysates were incubated with 25 µL of pre-cleared rabbit anti-HA Ab conjugated beads (EZview^TM^ Red anti-HA affinity gel, Sigma, USA) or with 25 µL protein G-Sepharose (GE Healthcare) for 2 h at 4°C for immunoprecipitation. After four washes with lysis buffer, 25 µL of 2× reducing sodium dodecyl sulfate polyacrylamide loading dye was added to the beads, the lysates, and the supernatants. All of the samples were incubated at 95°C for 5 min, and subjected to western blotting (WB).

### Western blotting

Proteins were collected after addition of lysis buffer to the cells, and separated on a 12% Tris-glycine gel and transferred to a nitrocellulose membrane. After blocking with 4% BSA in Tris-buffered saline with Tween-20 (TBST) for 1 h, the membrane was incubated with primary Ab (1∶1000 dilution) overnight at 4°C. The membrane was then washed 3 times with TBST, and incubated with HRP-conjugated goat anti-rabbit secondary (1∶5000) or HRP-conjugated goat anti-mouse secondary (1∶5000) in TBST with 5% non-fat milk for 1 h at room temperature, followed by thorough washes with TBST. The signals were visualized with enhanced chemiluminescence-plus WB detection reagents (Pierce).

### ELISA of IL-15

HeLa cells (2×10^6^/mL per dish ) were seeded on 10 cm dishes (10 mL/dish). The cells were treated with either TNF (5 ng/mL) for 4 h, or BFA (2 μM) or LMB (20 ng/mL) for 2 h in DMEM medium without FBS. Medium was collected after either different treatments or overexpression of plasmids. The medium was concentrated by use of Amicon Ultra10 kD centrifugal filters (Millipore Co. Billerica, MA). Protein concentration was measured with a BCA protein assay kit (Pierce). The concentrations of IL-15 from medium were determined by IL-15 ELISA kit (Invitrogen), according to the manufacturer's protocol. The linear range of the detection was 0–25,000 pg/mL.

### Statistical analyses

Means are presented with their standard errors. To determine the difference among groups, the data were analyzed by analysis of variance (ANOVA) followed by Tukey's multiple comparison test. To determine co-localization of IL-15 or IL-15Rα with organelle markers, Pearson's and Manders' correlation analyses were performed.

## Results

### TNF induces IL-15Rα and IL-15 nuclear export in a time-dependent manner

**Figure 1:**
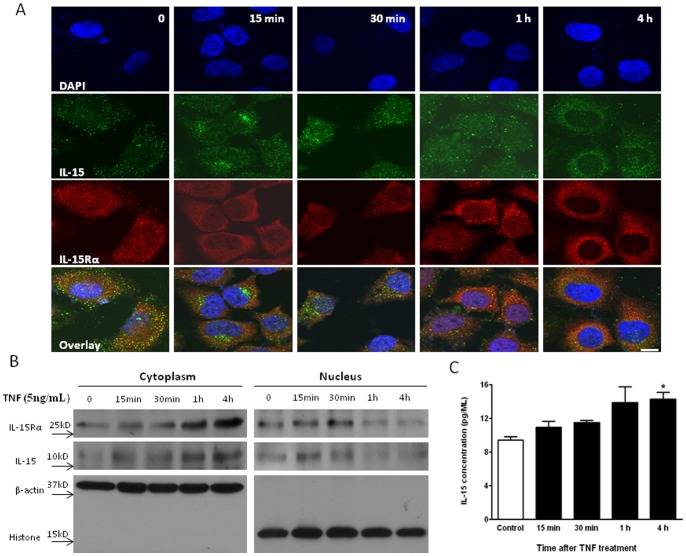
Time course of IL-15 and IL-15Rα nuclear export in HeLa cells treated with TNF. (A) ICC of IL-15 (green) and IL-15Rα (red) distribution after stimulation by TNF (5 ng/mL) for 0, 15 min, 30 min, 1 h, or 4 h. DAPI stained nuclei are shown in blue. Scale bar: 10 μm. (B) WB for IL-15 and IL-15Rα in the nucleus and cytoplasm at 0, 15 min, 30 min, 1 h, and 4 h after TNF treatment. Histone and actin were the respective markers used to show efficient separation of nucleus and cytoplasm. (C) IL-15 level in culture media of basal and TNF-stimulated cells as quantified by ELISA. *: p<0.05 from the non-stimulated control.

Under basal conditions, IL-15Rα was present both in the nucleus and cytoplasm. After treatment with TNF for 15 min, 30 min, 1 h, or 4 h, there was a time-dependent increase of IL-15Rα immunofluorescence in the cytoplasm and concurrent reduction in the nucleus. After 4 h of TNF treatment, most IL-15Rα was located in the cytoplasm. This suggests a predominant nuclear-to-cytoplasmic shuttling of IL-15Rα (Fig. 1A). The basal level of IL-15 protein expression was low. TNF treatment also induced a time-dependent increase of IL-15 immunofluorescence, initially seen mainly in the nucleus, but later was more pronounced in the cytoplasm. After 4 h of TNF treatment, most IL-15 was seen in the cytoplasm rather than the nucleus.

Consistently, WB showed a time-dependent increase of IL-15 and IL-15Rα protein in the cytoplasmic fraction, and a corresponding decrease in the nuclear fraction after TNF treatment (Fig. 1B). Furthermore, ELISA showed a significant increase of IL-15 in the medium at 4 h, though not at earlier times (Fig. 1C). Overall, the results show that TNF changed the dynamics of IL-15 and IL-15Rα trafficking, with nuclear export of both the ligand and its receptor and facilitated IL-15 secretion.

### Nuclear export of Il-15Rα and IL-15 is CRM1-mediated and LMB-sensitive

**Figure 2:**
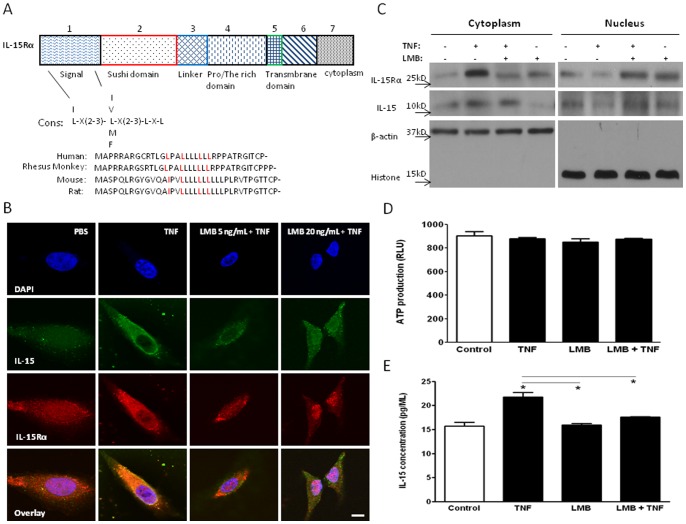
IL-15Rα and IL-15 undergo CRM1-mediated nuclear export. (A) Mapping of the IL-15Rα sequence showing putative NES interacting sites, with comparison of the putative NES in IL-15Rα with the consensus sequence (cons) of leucine-rich NES in IL-15 Rα from human and other species. (B) Nuclear export of IL-15 (green) and IL-15Rα (red) was inhibited by LMB after TNF induction in a dose-dependent manner. IL-15Rα was located in both nucleus and cytoplasm before TNF treatment. After TNF stimulation for 4 h, IL-15Rα and IL-15 shuttled from nucleus to the cytoplasm. LMB (5 or 20 ng/mL) co-treatment in the last 2 h prevented the export of IL-15Rα and IL-15. DAPI stained nuclei are shown in blue. Bar: 10 μm. (C) WB of IL-15 and IL-15Rα in the nucleus and cytoplasm after vehicle, TNF (5 ng/mL), LMB (20 ng/mL), or combined treatment. The markers actin and histone were used to show efficient separation of nucleus and cytoplasm. (D) Cell viability in the matching treatment groups shown by ATP production. (E) IL-15 level in culture media of basal or stimulated cells, as quantified by ELISA. *: p<0.05 from the non-stimulated control.

The sequence of human IL-15Rα contains a leucine-rich area at the N-terminus, consistent with that of the NES responsible for interaction with CRM1 (exportin 1). Specifically, NES is a short sequence of amino acids consisting of leucine (or a hydrophobic amino acid) spaced by two to three lengths of amino acids, which CRM1 recognizes. This particular sequence of IL-15Rα is highly conserved among species, including human beings, rhesus monkey, rat, and mouse (Fig. 2A). To test the hypothesis that TNF facilitates nuclear export of IL-15Rα via the CRM1 system, we treated the cells with LMB, a specific inhibitor of the NES-dependent nuclear export receptor CRM1. HeLa cells without treatment served as negative controls whereas those treated with TNF for 4 h served as positive controls. In the experimental groups, LMB was added at either 5 or 20 ng/mL for 2 h in addition to the TNF treatment for 2 h before addition of LMB. In comparison with the cells treated with TNF for a total of 4 h and vehicle for the last 2 h, the LMB-treated groups showed that IL-15 and IL-15Rα remained in the nucleus. The effect was more pronounced in cells receiving 20 ng/mL of LMB than in those receiving 5 ng/mL, suggesting a dose-dependent effect of LMB to block IL15-Rα nuclear export (Fig. 2B).

To further verify the nuclear export of IL-15 and its specific receptor in response to TNF and the involvement of CRM1, we performed WB on subcellular fractions of HeLa cells treated with vehicle, 5 ng/mL of TNF, 20 ng/mL of LMB, or both. The effectiveness of subcellular fractionation was confirmed by the probing of actin and histone, which showed predominant presence in the cytoplasm and nucleus, respectively. In the cytoplasmic fraction, TNF treatment increased IL-15 and IL-15Rα protein. This was blocked by LMB, with or without TNF co-treatment. In the nuclear fraction, TNF treatment decreased IL-15 and IL-15Rα; this was also reversed by LMB, with or without TNF co-treatment (Fig. 2C). In none of the conditions was cell viability compromised (Fig. 2D). In concentrated cell culture medium, TNF treatment for 4 h also increased IL-15 levels, whereas LMB co-treatment attenuated this increase (Fig. 2E).

### IL-15 and IL-15Rα show differential cytoplasmic trafficking

**Figure 3:**
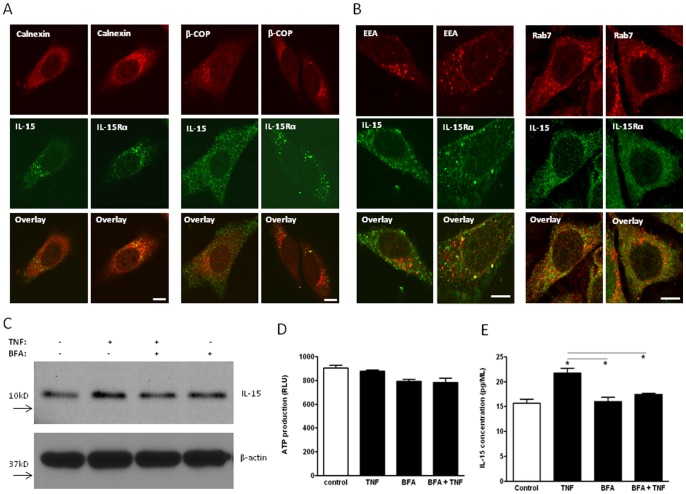
Intracellular trafficking of IL-15 and IL-15Rα. (A and B) Distribution of IL-15 and IL-15Rα in intracellular vesicles. After TNF treatment for 4 h, IL-15 showed partial co-localization with late endosomes but very little with other organelles tested. IL-15Rα showed co-localization with both early and late endosomes, and to a lesser extent with endoplasmic reticulum and the Golgi complex. (C) WB showing IL-15 levels in the cell lysate after TNF (5 ng/mL) or BFA (2 μM) treatment. (D) Cell viability after TNF or LMB treatment shown by ATP production. (E) ELISA of cell culture medium showing IL-15 level in response to TNF or BFA treatment. *: p<0.05 from the non-stimulated control.

It has been shown that overexpressed long signaling peptide (LSP)-IL-15-GFP is seen in the early endosomes [10]. To test the hypothesis that endogenous IL-15 uses similar routes as the overexpressed fusion protein, we determined the co-localization of IL-15 or IL-15Rα immunoreactivity with organelle markers 4 h after TNF treatment, and performed confocal microscopic analysis of potential co-localization. IL-15 showed partial co-localization with late endosomes but very little with other organelles tested. IL-15Rα showed co-localization with both early and late endosomes, as well as endoplasmic reticulum and Golgi, although to a lesser extent (Fig. 3A–B). Quantification was performed by use of the NIH Image J program with JACoP plug-in (Table 1). Using Pearson's coefficient that estimates the correlation of intensity distribution between two proteins of interest [23], we identified the correlations of EEA with IL-15 (0.319) and IL-15Rα (0.355) in the cells 4 h after TNF treatment. At this time point, there was a greater association of IL-15 (0.398) and IL15-Rα (0.516) with Rab7. The higher Pearson's coefficient for Rab7 than EEA suggests that more IL-15 and IL-15Rα were present in late endosomes than early endosomes 4 h after TNF treatment. Manders' coefficients, which provide information of the proportion of the detected signal representing one protein coincident with the signal representing another protein, showed that the level of co-localization of Rab7 with IL-15 (M1: 0.257, M2: 0.271) and IL-15Rα (M1: 0.224, M2: 0.269) were similar. However, the co-localization of EEA with IL-15 and IL-15Rα showed spatial variation. Only 4.2% of IL-15 was present in EEA, while 13.2% of EEA overlapped with IL-15. By contrast, 15.0% of IL-15Rα was localized in EEA, whereas 24.4% of EEA showed IL-15Rα immunoreactivity (Table 1). Thus, there was more EEA with IL-15Rα than EEA with IL-15, suggesting that the ligand and receptor did not have close association with each other at this time. Moreover, the trafficking of endogenous IL-15 was at least partially sorted to secretory pathways, suggesting possible involvement of its LSP sequence. IL-15 and IL-15Rα apparently did not use the same trafficking route in the cytoplasm, nor did they follow the same kinetics of intracellular sorting.

**Table 1:** Co-localization analysis.

	EEA			Rab7		
	Pearson's Coefficient	Manders' Coefficients		Pearson's Coefficient	Manders' Coefficients	
		M1	M2		M1	M2
IL15	0.319	0.042	0.132	0.398	0.257	0.271
IL15Rα	0.355	0.150	0.244	0.516	0.224	0.269

M1: fraction of green fluorescence (IL15 or IL15Rα) overlapping with red fluorescence (EEA or Rab7).

M2: fraction of red fluorescence (EEA or Rab7) overlapping with green fluorescence (IL15 or IL15Rα).

The role of early and late endosomes in cytokine secretion is unclear, but endocytotic recycling of IL-15 after its exocytosis remains possible. We previously have shown a rapid, time-dependent endocytosis of fluorescently conjugated (exogenous) IL-15 that is associated with IL-15Rα during the endocytosis by cerebral endothelial cells [24]. Here, TNF treatment for 4 h did not induce endogenous IL-15 trafficking to early endosomes. BFA, a blocker for ER to Golgi trafficking, had no effect in increasing cellular IL-15 in WB (Fig. 3C). However, there was a reduction of soluble IL-15 recovered from cell culture medium shown by ELISA (Fig. 3E). None of the treatments (TNF, BFA, or both) reduced cell viability or ATP production (Fig. 3D). Overall, the reduced IL-15 concentration in the medium after BFA indicates that ER-to-Golgi trafficking is involved in TNF-facilitated secretion of IL-15.

### ARF6 facilitates IL-15Rα trafficking

**Figure 4:**
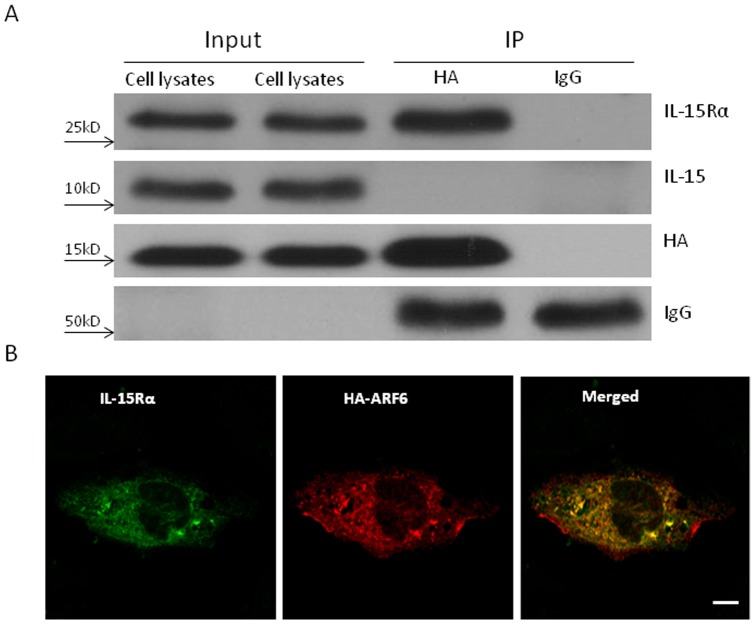
ARF6 interacts with IL-15Rα. (A) ARF6 was bound to IL-15Rα. Cells were transfected with ARF6-HA 24 h earlier. ARF6 protein was immunoprecipitated and subjected to WB for IL-15 and IL-15Rα. Immunoprecipitation with nonspecific IgGs was done as the control. (B) ARF6 co-localized with IL-15Rα in the plasma membrane and some organelles. After co-transfection, ARF6-HA (green) and IL-15Rα (red) were co-localized, as shown by confocal microscopy. Bar: 10 μm.

To test the hypothesis that the small GTPase ARF6 promotes IL-15 exocytosis, the cells were transfected with HA-ARF6 fusion protein plasmids, treated with TNF or PBS for 4 h (n = 3/group), and subjected to cross-linking with 1% PFA to capture the transient and non-covalent interactions between ARF6 and IL-15/IL-15Rα. ARF6 was immunoprecipitated with an anti-HA antibody, as HA is fused to the N-terminus of ARF6. To determine whether ARF was associated with IL-15 or IL15-Rα, WB for IL-15 and IL-15Rα was performed on the precipitate. In the whole cell lysate (control fraction), both IL-15Rα and ARF6 immunoreactivity was present. In the ARF6-HA pull-down immunoprecipitate, there was increased signal intensity of both IL-15Rα and HA greater than that seen in the whole cell lysate when an equal amount (25 µL) of protein lysate was loaded for WB (Fig. 4A). By contrast, IL-15 immunoreactivity did not show an increase (Fig. 4A). The results suggest that IL-15Rα was bound to ARF6 whereas IL-15 did not have direct interaction with ARF6 at this time.

In cells co-transfected with IL-15Rα-GFP and ARF6-HA plasmids 24 h earlier, confocal microscopic analysis showed that IL-15Rα co-localized with ARF6 in the plasma membrane and intracellular organelles (Fig. 4B). This further supports an interaction of ARF6 with IL-15Rα that might be involved in the exocytosis trafficking of IL-15.

### Involvement of ARF6 in IL-15 secretion is shown by inhibited secretion in cells transfected with dominant negative ARF6

**Figure 5:**
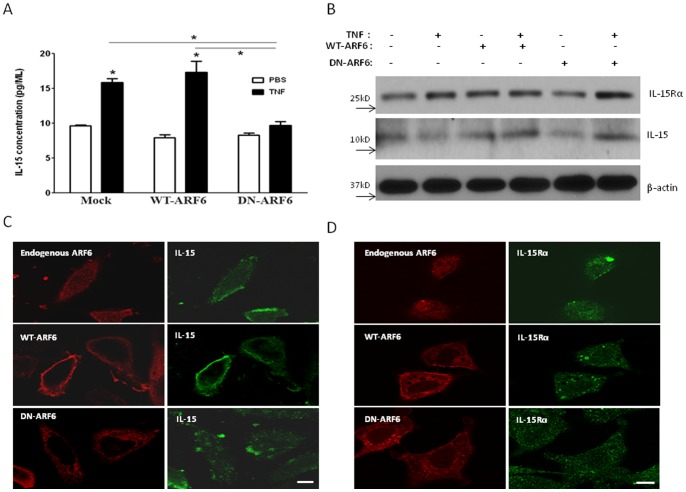
ARF6 localizes to plasma membrane and endosomes and it is crucial to IL-15 secretion after TNF treatment. HeLa cells were transfected with pCDNA3.1 (control), WT-ARF6, or DN-ARF6 plasmids 48 h before the assay. (A) In control cells and those overexpressing WT-ARF6, TNF treatment for 4 h increased IL-15 release. The cells overexpressing DN-ARF6 did not show a response. In none of the groups did PBS vehicle treatment increase IL-15 release. Data are representative of three independent experiments. *: p<0.05 compared with the control. (B) The overexpression of WT-ARF6 and/or TNF treatment seemed to increase the expression of IL-15 and IL-15Rα, shown by WB of whole cell lysates. (C) The potential interaction of ARF6 with IL-15 was shown by ICC. The left panel is ARF6 immunoreactivity (red, stained with anti-ARF6 in control cells and anti-HA in cells overexpressing ARF6) and the right panel is IL-15 immunoreactivity (green). In control cells, endogenous ARF6 showed a diffuse vesicular pattern of cytoplasmic distribution. In cells overexpressing WT-ARF6 that increased overall expression as well as cell surface distribution of ARF6, IL-15 signal was also increased and showed cell surface distribution co-localizing with WT-ARF6. In cells overexpressing DN-ARF6, the small increase of ARF6 signal remained cytoplasmic, and did not show co-localization with IL-15. Bar: 10 μm. D) Intracellular distribution of IL-15 Rα (green) did not show co-localization with endogenous ARF6 in control cells (stained with an ARF6 Ab, red) or in those overexpressing WT-ARF6 (middle panel) or DN-ARF6 (lower panel, HA Ab staining, red). Bar: 10 μm.

Expression of either WT- or DN-ARF6 did not affect the basal release of IL-15 (Fig. 5A). After 4 h of TNF stimulation, IL-15 secretion was increased. However, the DN-ARF6 transfected group did not show a corresponding increase in response to TNF. The difference between the DN-ARF6 group and either the mock-transfected or WT-ARF6 groups was significant. Compared with the mock control group without ARF6 overexpression, IL-15 secretion in DN-ARF6-expressing cells was decreased by 39% (n = 3/group). As this may represent either accelerated secretion or increased production of IL-15, we further determined the effect of ARF6 expression on IL-15 immunoreactivity in the presence of TNF treatment, and compared the patterns with those of IL-15Rα. Like TNF treatment (lane 2), WT-ARF6 appeared to have a minor effect to increase IL-15 and IL-15Rα (lane 3), and co-treatment had a greater effect to increase IL-15. DN-ARF6 did not show such effect, but TNF treatment of cells transfected with DN-ARF6 persistently increased IL-15 as well as IL-15Rα (Fig. 5B). In cells transfected with WT-ARF6, the HA immunofluorescence representing ARF6 was present at the cell surface as well as cytoplasm, and was co-localized with IL-15 immunofluorescence that was increased compared with the non-transfected cells. By contrast, DN-ARF6 showed an intracellular location and did not show co-localization with IL-15 (Fig. 5C). IL-15Rα, by comparison, did not show clear co-localization with either WT-ARF6 or DN-ARF6 (Fig. 5D). Thus, WT-ARF6 overexpression mainly acted to facilitate IL-15 sorting to the cell surface and probably its subsequent exocytosis.

## Discussion

Here we showed that TNF induced nuclear export of IL-15 and IL-15Rα in a process involving CRM1, directed them to diverse intracellular routes, and helped exocytotic secretion of IL-15 that was facilitated by ARF6. This is the first demonstration that CRM1 and ARF6 participate in the regulated production/redistribution of IL-15 and its specific receptor. The results may have broad implications in deciphering the inflammatory cascade within cells.

The nuclear localization of IL-15, similar to that reported in MELREO melanoma cells [25], is probably mediated by its specific receptor IL-15Rα, as IL-15 does not appear to be able to enter the nucleus by itself [10]. TNF treatment showed a time-dependent effect to decrease nuclear IL-15Rα and IL-15 and increase their cytoplasmic content. This poses several important questions: (a) Does IL-15 get out of the nucleus along with its receptor? (b) What are the mediating mechanisms by which TNF induces such changes? and (c) Does the effect of TNF mainly lie in the prevention of nuclear entry of newly synthesized proteins, or facilitation of nuclear transport by redistribution of the IL-15 and receptor complex from nucleus back to cytoplasm?

To cross-validate the ICC findings of nuclear export of IL-15 and its specific receptor, we performed subcellular fractionation. Short-term stimulation (15 min to 4 h) was sufficient to reduce nuclear IL-15 and increase cytoplasmic IL-15 and its specific receptor. This suggests that nuclear export and redistribution of IL-15 and IL-15Rα is a more likely possibility than *de novo* synthesis along with blocked nuclear entry. In response to a proinflammatory stimulus like TNF, there was an efficient and rapid recruitment of the IL-15 system. Furthermore, TNF increased the secretion of IL-15 by 4 h, implying that IL-15 may exert endocrine as well as autocrine and juxtacrine functions.

Like other proteins capable of nuclear export, IL-15Rα has a consensus NES sequence with a leucine-rich domain at the N-terminus, enabling IL-15Rα nuclear export. IL-15 probably remained associated with IL-15Rα so that it was also nuclear exported in these studies. The involvement of NES in the nuclear export of IL-15 and IL-15Rα was further supported by the inhibitory effect of LMB, which interacted with the NES-dependent nuclear export receptor CRM1 [26]. A specific effect of TNF on CRM1 has been shown in several other systems. In hepatocytes, TNF modulates the nuclear location of C/EBPβ and its phosphorylation at Ser^239^, a process involved in oxidative stress and cancer-related cachexia [27]. It has been shown that TNF acts through TNF receptor-associated factor 2 (TRAF2) to interact with inhibitors of apoptosis cIAP1 and cIAP2 and inhibit caspase activities [28]. IL-15Rα can also act through TRAF2 to regulate activation of the nuclear factor (NF)-κB pathway [10], [29]. Thus, TNF might act through TRAF2 to induce interactions with CRM1 and IL-15Rα, thus facilitating the nuclear export of the IL-15/IL-15Rα complex.

IL-15 and IL-15Rα both have complex intracellular trafficking patterns [30]. For IL-15, transcription by the 21 amino acid LSP directs it to non-secretory organelles whereas that by the 48 amino acid LSP may guide it to the secretory apparatus [31]. We show here that endogenous IL-15 was at least partially located in the late endosomes 4 h after TNF stimulation. There was little in early endosomes, and no apparent presence in the ER or Golgi. In contrast with IL-15, IL-15Rα showed a somewhat different intracellular organelle distribution. This at least illustrates different kinetics of trafficking of IL-15 and its receptor, and indicates dissociation of these two in later stages of cytoplasmic trafficking. Though we did not differentiate IL-15Rα isoforms, many of them co-exist in cells and respond to TNF treatment with STAT3 activation [21] as well as other signaling events. Altogether, these events may alter the fate of IL-15 in the cells.

Small GTP-binding proteins play important roles in vesicular transport. ARF proteins are GTPases that function as regulators of membrane traffic [32]. ARF6 has two bound conformations: GTP-bound and active, and GDP-bound and inactive. ARF6 activation is mediated by guanine nucleotide exchange factors (GEFs) that exchange GTP for GDP, whereas downregulation of ARF6 activity is mediated by GTPase activating proteins (GAPs) with hydrolysis of GTP to GDP. As ARF6 cycles through its active and inactive conformations, it facilitates ligand internalization at the cell surface, subsequent trafficking along the endocytic pathway, endosomal recycling, and fusion of the endosomal membrane with the plasma membrane. Distinct effector molecules determine the outcome of ARF6 activity with GTP/GDP cycling, and destine the ligands to discrete subcellular locations [33]. ARF6 is frequently involved in the trafficking of biological membranes and transmembrane protein cargos [34]. Our results show that ARF6 may not have direct association with IL-15, but it does bind to IL-15Rα. This binding might have contributed to the increased IL-15 secretion, since DN-ARF6 inhibited the exocytosis of IL-15.

In summary, we showed that TNF can induce nuclear export of IL-15 and IL-15Rα in a time-dependent manner, a process that was essentially complete by 4 h. Inhibition of this process by LMB indicates that CRM1 was a crucial mediator of the nuclear export. There were different kinetics of intracellular trafficking of IL-15 and IL-15Rα, and ARF6 specifically interacted with IL-15Rα and facilitated IL-15 exocytosis. The novel cellular mechanisms mediating IL-15 nuclear export and secretion in response to TNF may have broad clinical implications for inflammatory diseases and cancer.
